# The protective role of hearing aids on delayed recall and spatial orientation in age-related hearing loss

**DOI:** 10.3389/fpubh.2026.1753247

**Published:** 2026-03-09

**Authors:** Jiacheng Wang, Minqian Gao, Yiwen Luo, Haidi Yang, Ling Chen

**Affiliations:** 1 School of Medical Technology and Information Engineering, Zhejiang Chinese Medical University, Hangzhou, China; 2 Department of Otolaryngology, Sun Yat-sen Memorial Hospital, Sun Yat-sen University, Guangzhou, China

**Keywords:** age-related hearing loss, delayed recall, hearing aid, protective, spatial orientation

## Abstract

**Background and objective:**

Age-related hearing loss (ARHL) is a significant risk factor for cognitive decline that can be mitigated using hearing aids (HAs). This study investigated the impact of HAs on cognitive function in ARHL, specifically targeting delayed recall and spatial orientation, and identified the influencing factors using multivariate logistic regression.

**Methods:**

This is a cross-sectional study. A total of 104 ARHL patients from July 2023 to October 2025 were enrolled, dividing them into HA users (HA+ group, *n* = 47) and non-HA (HA− group, *n* = 57). The patients underwent audiological and cognitive assessments, including pure tone average (PTA), Montreal Cognitive Assessment (MoCA), and Mini-Mental State Examination (MMSE). Group comparisons were performed using these measures. Logistic regression was used to identify predictors of impaired delayed recall and spatial orientation, considering variables such as age, sex, HA+/− status, education, severity of hearing loss, depressive symptoms, living alone, smoking, alcohol consumption, diabetes, and hypertension.

**Results:**

The HA + group had significantly better MoCA (24.49 ± 2.78 vs. 21.00 ± 3.63; *Z* = −4.881, *p* < 0.001) and MMSE (25.81 ± 2.45 vs. 23.33 ± 3.33; *Z* = −4.118, *p* < 0.001) scores than the HA- group, notably in delayed recall (*Z* = −2.653, *p* = 0.008) and spatial orientation (*Z* = −3.643, *p* < 0.001). HAs were a significant protective factor for both delayed recall [OR = 0.271, 95% confidence interval (CI) = (0.080, 0.914)] and spatial orientation [OR = 0.233, 95% CI = (0.066, 0.823)]. Additionally, higher educational levels were associated with better cognitive function.

**Discussion:**

HAs improved cognitive function in patients with ARHL, especially in terms of delayed recall and spatial orientation, with education offering additional protection.

## 1 Introduction

In 2016, age-related hearing loss (ARHL) ranked among the top three leading causes of years lived with disability (YLDs), with a prevalence of 1.27 billion patients [95% uncertainty interval (UI) = (1.21, 1.34)] (1). In 2019, 62.1% [95% UI = (60.2, 63.9)] of people with hearing loss (HL) were over the age of 50, and projections indicate that this number will reach 2.45 billion [95% UI = (2.35, 2.56)] by 2050 (2). In 2024, The Lancet Commission reported that ARHL is associated with an increased risk of cognitive decline or dementia, with a hazard ratio (HR) of 1.37 [95% confidence interval (CI) = (1.00, 1.87)] (3). This condition accounted for approximately 7% of the population-attributable fraction (PAF) of potentially modifiable risk factors for dementia, with a relative risk (RR) of 1.4 [95% CI = (1.0, 1.9)].

Longitudinal studies indicated that ARHL accelerated global cognitive aging, particularly affecting delayed recall and spatial orientation. HL was linked to poorer global cognitive performance over 7 years [*β* = −0.11, 95%CI = (−0.22, 0.01)], equating to approximately 4.6 years of aging, and notably impaired delayed recall [*β* = −0.10, 95%CI = (−0.21, 0.02)] (4). Additionally, ARHL increased postural sway during visual disturbances, suggesting deficits in multisensory integration and a higher risk of imbalance or falls (5). On the other hand, previous research indicated that delayed recall and spatial orientation tend to deteriorate earlier in the progression of cognitive aging, with observable alterations manifesting from midlife or even earlier (6). These cognitive domains exhibited more pronounced age-related declines compared to many other cognitive areas.

The Commission further demonstrated that the use of hearing aids (HAs) is protective against dementia and mitigates cognitive deterioration, as evidenced by a significantly lower risk of cognitive decline [HR = −1.32, 95% CI = (−3.34, 0.71)]. A meta-analysis corroborated these findings, revealing that HA users had a significantly reduced risk of cognitive decline [HR = 0.81, 95% CI = (0.76, 0.87)] and experienced 3% improvement in cognitive test scores following the use of hearing rehabilitation devices (7). Our prior research also demonstrated that HAs can significantly augment cognitive reserve in ARHL, consequently enhancing cognitive function (8).

However, the cognitive benefits of hearing intervention are still debated. A recent multicenter randomized controlled trial (RCT) showed no significant cognitive improvement over 3 years from hearing intervention in the primary analysis, but a sensitivity analysis revealed that using HAs for an average of 7.2 h daily reduced cognitive decline by 48% (9). These results highlighted that cognitive changes in ARHL were influenced by various factors such as gender, education, alcohol consumption, and HL severity, not just HA use.

While evidence suggested HAs offer cognitive benefits, a majority of research emphasized overall cognitive scores rather than specific cognitive domains. However, general cognitive assessments may not possess adequate sensitivity to identify the potential cognitive benefits associated with HA use that manifest in the early stages of age-related cognitive decline. Therefore, focusing on specific cognitive domains, such as delayed recall and spatial orientation, may offer a more precise method of detecting the initial cognitive effects of HA use. Some studies showed that HAs improved certain functions such as delayed recall [95% CI = (−1.2, −0.05)] and visual memory [95%CI = (−0.9, −0.15)] (10). Additionally, HAs enhanced postural stability and reduced dependence on somatosensory input for balance when visual and somatosensory cues were limited, indicating their impact extended beyond hearing to broader cognitive and sensorimotor networks (11, 12).

This study examined how HAs affect various cognitive domains in ARHL using logistic regression to adjust for these factors, especially in delayed recall and spatial orientation. These findings offer clinical insights into the role of HAs in preserving cognitive function in ARHL patients.

## 2 Methods

### 2.1 Patients

**Figure 1:**
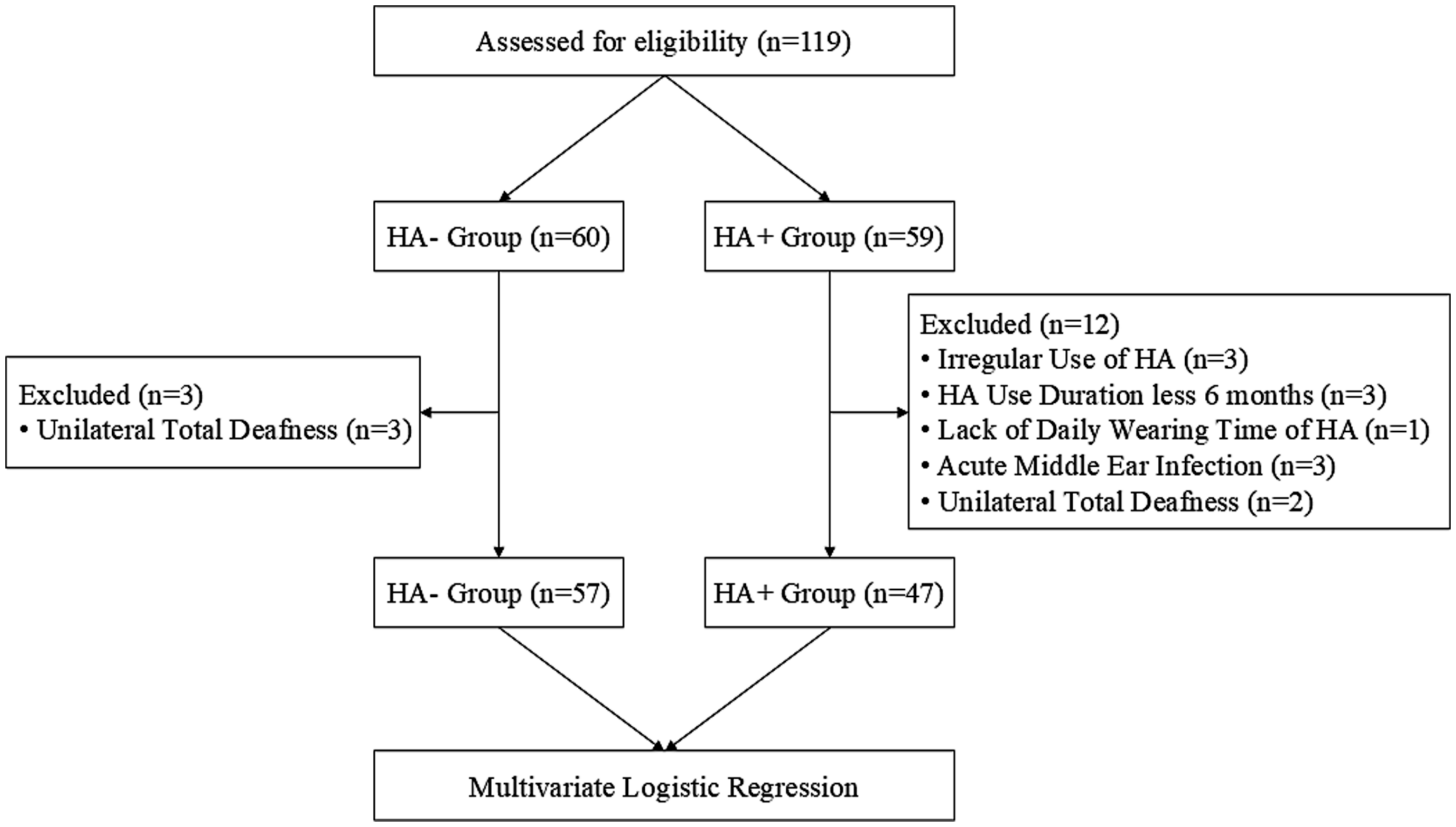
Flow diagram.

Patients diagnosed with ARHL were recruited for this study between July 2023 and October 2025 at the Sun Yat-sen Memorial Hospital, Sun Yat-sen University. Patients were enrolled according to the following inclusion criteria: (1) age 50–90 years, (2) native Chinese-language speaker, and (3) right-handed. The exclusion criteria were as follows: (1) acute middle ear infection, (2) any severe neurological and psychological disorders and dementia, and (3) missing cognitive or audiological data. All patients provided written informed consent and completed audiological and cognitive assessments, including the pure-tone audiometry test, Montreal Cognitive Assessment (MoCA) and its sub-items, Mini-Mental State Examination (MMSE) and its sub-items, Geriatric Depression Scale-15 (GDS-15), Hamilton Depression Scale-24 (HAMD-24), and other demographic data. All procedures were approved by the Ethical and Scientific Committee of the Sun Yat-sen Memorial Hospital, Sun Yat-sen University. ARHL patients were divided into two groups: (1) HA-: ARHL patients without HA and (2) HA+: ARHL patients who were fitted with HAs for at least 6 months. The self-reported HA usage averaged 4.03 ± 3.90 years and 7.19 ± 3.16 h daily (Figure 1).

### 2.2 Audiological and cognitive assessment

For the audiological assessment, pure-tone hearing thresholds were measured using pure-tone audiometry with an AC40 diagnostic audiometer (Interacoustics, Denmark) in a standard soundproof room (GB/T 16296.1–2018). The evaluation was conducted for each ear, covering a frequency range of 0.125 to 8.0 kHz for air conduction and 0.25 to 4.0 kHz for bone conduction, respectively. The hearing thresholds of 0.5, 1.0, 2.0, and 4.0 kHz were averaged to calculate the pure tone average (PTA). According to 2021 World Health Organization (WHO), HL severity was defined as mild (PTA = 20–34 dB HL), moderate (PTA = 35–49 dB HL), moderate–severe (PTA = 50–64 dB HL), severe (PTA = 65–79 dB HL), or profound (PTA = 80–94 dB HL).

Cognitive function was measured using the MoCA and MMSE scores. The MoCA is scored from 0 to 30 and contains 7 sub-items, including visuospatial and executive function, naming, attention, language, abstraction, delayed recall, and orientation. The MoCA score was adjusted for patients’ education ≤ 12 years. The adjusted MoCA was analyzed in this study. The MMSE is also scored from 0 to 30 and comprises 6 sub-items: temporal orientation, spatial orientation, registration, attention and calculation, recall, language and praxis. In this study, cognitive impairment was defined as MoCA < 26, while depressive symptoms were defined as HAMD-24 > 8.

### 2.3 Statistical analysis

Statistical analysis and visualization were performed using R (Ver.4.4.2). The Shapiro–Wilk test was used to evaluate the normality, indicating that MoCA and its sub-items, MMSE and its sub-items, as well as bilateral PTA, did not follow a normal distribution in both HA + and HA- groups. Mann–Whitney U-tests assessed differences in MoCA and its sub-items, MMSE and its sub-items, and bilateral PTA between groups. The *χ*^2^ test was used to evaluate age, sex, education, HL severity, living alone, smoking status, alcohol consumption, diabetes, hypertension, and depressive symptoms.

Multivariate logistic regression modeling was used to estimate the strength of the association between independent variables and cognitive impairment (delayed recall and spatial orientation). The logistic models were constructed using four models. Model 1 was unadjusted for the relationship between HA (+/−), sex, education, severity of HL, hypertension, and cognitive impairment. Model 2 was adjusted for age. Model 3 was additionally adjusted for living habits, including living alone, smoking status, and alcohol consumption. Model 4 was fully adjusted for age, living habits, and chronic diseases such as diabetes and depressive symptoms. Odds ratios (ORs) and 95% CI were calculated for all logistic models. In this study, statistical significance was set at a *p*-value of < 0.05.

## 3 Result

This study included 104 patients, 50 females (48.08%) and 54 males (51.92%), with 47 and 57 patients in the HA + and HA − groups, respectively. The average age was 69.84 ± 8.69 years, 68.83 ± 9.37 years in the HA + group, and 70.67 ± 8.09 years in the HA- group, while the average education was 10.81 ± 3.81 years, 11.68 ± 3.95 years in the HA+ group, and 10.09 ± 3.57 years in the HA- group. Demographic and clinical characteristics between the HA+ and HA− groups are presented; age and education were not significantly different between groups, and the HA− group showed fewer males than the HA + group (Table 1).

**Table 1:** Demographic and clinical characteristics between HA+/− groups (*n*, %).

Characteristics/Groups	HA+ (*n* = 47)	HA− (*n* = 57)	*χ* ^2^	*p*
Age				
50 ~ 59	7 (14.89)	4 (7.02)	0.2117	0.549
60 ~ 69	17 (36.17)	19 (33.33)		
70 ~ 79	16 (34.05)	24 (42.11)		
80 ~ 89	7 (14.89)	10 (17.54)		
Gender				
Female	16 (34.04)	34 (59.65)	6.766	0.009*
Male	31 (65.96)	23 (40.35)		
Education				
Primary School	6 (12.77)	14 (24.56)	7.041	0.071
Middle School	13 (27.66)	15 (26.32)		
High School or GED	11 (23.40)	19 (33.33)		
College or above	17 (36.17)	9 (15.79)		
Hearing loss severity				
Mild + Moderate	4 (8.51)	0	26.922	<0.001*
Moderated Severe	17 (36.17)	46 (80.70)		
Severe	17 (36.17)	11 (19.30)		
Profound	9 (19.15)	0		
Smoking	9 (19.15)	12 (21.05)	0.058	0.810
Alcohol	6 (12.77)	7 (12.28)	0.006	0.941
Hypertension	18 (38.30)	24 (42.11)	0.155	0.694
Diabetes	11 (23.40)	5 (8.77)	4.237	0.040*
Depression	6 (12.77)	10 (17.54)	0.452	0.502

HA+, age-related hearing loss with hearing aid; HA−, age-related hearing loss without hearing aid; GED, general equivalency diploma; *p < 0.05.

### 3.1 HA + showed better cognition despite worse hearing

The average MoCA was 24.49 ± 2.78 in the HA + group, significantly better than 21.00 ± 3.63 in the HA- group (*Z* = −4.881, *p* < 0.001), especially in delayed recall (*Z* = −2.653, *p* = 0.008), visuospatial and executive function (*Z* = −3.566, *p* < 0.001), language (*Z* = −2.637, *p* = 0.008), attention (*Z* = −2.478, *p* = 0.013), naming (*Z* = −2.776, *p* = 0.005), and abstraction (*Z* = −3.138, *p* = 0.002).

The average MMSE was 25.81 ± 2.45 in the HA + group, significantly better than 23.33 ± 3.33 in the HA- group (*Z* = −4.118, *p* < 0.001), especially in spatial orientation (*Z* = −3.643, *p* < 0.001), recall (*Z* = −2.629, *p* = 0.009), registration (*Z* = −2.352, *p* = 0.019), attention and calculation (*Z* = −2.417, *p* = 0.016), and language and praxis (*Z* = −2.795, *p* = 0.005).

In the MoCA test, orientation was similar between the HA + and HA− groups (*p* = 0.051). In the MMSE test, the HA + group had better spatial orientation, while temporal orientation was similar between both groups (*p* = 0.605). Comprehensive results are provided in Supplementary Table 1.

However, the bilateral PTA was 66.15 ± 13.69 dB HL in the HA + group, significantly worse than 58.43 ± 6.60 dB HL in the HA- group (*Z* = −3.561, *p* < 0.001).

### 3.2 HA and higher education protect for delayed recall

In the unadjusted model (Model 1), HA [OR = 0.271, 95% CI = (0.084, 0.875), *p* = 0.029] and a higher educational level [college or above, OR = 0.057, CI = (0.009, 0.343), *p* = 0.002] were protective factors for delayed recall. In contrast, male sex [OR = 4.012, 95% CI = (1.301, 12.376), *p* = 0.016] was significantly associated with a higher risk of delayed recall impairment.

These associations remained statistically significant after adjusting for all covariates in Model 4, with HA [OR = 0.271, 95% CI = (0.080, 0.914), *p* = 0.035] and higher education [college or above, OR = 0.037, 95% CI = (0.005, 0.269), *p* = 0.001] continuing to show protective effects, while male sex [OR = 6.779, CI = (1.563, 29.407), *p* = 0.011] remained a risk factor for delayed recall (Table 2).

**Table 2:** Logistic regression analysis of delayed recall in age-related hearing loss.

Variable	Model 1		Model 2		Model 3		Model 4	
	OR (95% CI)	*p*	OR (95% CI)	*p*	OR (95% CI)	*p*	OR (95% CI)	*p*
Group								
Hearing aids (−)	Reference		Reference		Reference		Reference	
Hearing aids (+)	0.271 (0.084, 0.875)	0.029	0.278 (0.085, 0.907)	0.034	0.263 (0.080, 0.870)	0.029	0.271 (0.080, 0.914)	0.271
Gender								
Female	Reference		Reference		Reference		Reference	
Male	4.012 (1.301, 12.376)	0.016	3.975 (1.289, 12.262)	0.016	6.558 (1.555, 27.656)	0.010	6.779 (1.563, 29.407)	0.011
Education								
Primary school	Reference		Reference		Reference		Reference	
Middle school	0.155 (0.028, 0.850)	0.032	0.157 (0.029, 0.862)	0.033	0.154 (0.027, 0.872)	0.034	0.149 (0.026, 0.872)	0.035
High school or GED	0.541 (0.094, 3.105)	0.490	0.536 (0.093, 3.092)	0.485	0.493 (0.082, 2.967)	0.440	0.508 (0.084, 3.069)	0.460
College or above	0.057 (0.009, 0.343)	0.002	0.055 (0.009, 0.339)	0.002	0.038 (0.005, 0.266)	0.001	0.037 (0.005, 0.269)	0.001
Hearing loss								
Mild + moderate	Reference		Reference		Reference		Reference	
Moderated Severe	1.985 (0.143, 27.461)	0.609	1.953 (0.139, 27.463)	0.620	1.872 (0.145, 24.233)	0.631	1.911 (0.144, 25.394)	0.624
Severe	0.857 (0.063, 11.591)	0.907	0.837 (0.061, 11.531)	0.894	0.730 (0.058, 9.141)	0.807	0.740 (0.058, 9.512)	0.818
Profound	1.437 (0.087, 23.677)	0.800	1.393 (0.083, 23.401)	0.818	1.184 (0.075, 18.623)	0.905	1.136 (0.070, 18.547)	0.928
Hypertension								
Hypertension (−)	Reference		Reference		Reference		Reference	
Hypertension (+)	1.376 (0.496, 3.814)	0.540	1.008 (0.952, 1.067)	0.777	1.392 (0.475, 4.079)	0.546	1.373 (0.467, 4.040)	0.565

OR, odds ratio; CI, confidence interval; GED, general equivalency diploma.

Model 1, unadjusted; Model 2, Model 1 + only adjusted age; Model 3, Model 2 + adjusted for living habits, including living alone, smoking and alcohol status; Model 4, Model 3 + adjusted for chronic disease, including diabetes and depressive symptoms.

### 3.3 HA and higher education protect for spatial orientation

In the unadjusted model (Model 1), college and above education [OR = 0.125, CI = (0.026, 0.611), *p* = 0.010] were associated with a lower risk of spatial orientation.

After adjusting for all covariates in Model 4, HA [OR = 0.233, 95% CI = (0.066, 0.823), *p* = 0.024], and college or above education levels [OR = 0.101, 95% CI = (0.016, 0.619), *p* = 0.013] showed a protective effect on spatial orientation. Hypertension [OR = 2.914, 95% CI = (1.012, 8.396), *p* = 0.048] was significantly associated with a decline in spatial orientation (Table 3).

**Table 3:** Logistic regression analysis of spatial orientation in age-related hearing loss.

Variable	Model 1		Model 2		Model 3		Model 4	
	OR (95% CI)	*p*	OR (95% CI)	*p*	OR (95% CI)	*p*	OR (95% CI)	*p*
Group								
Hearing aids (+)	Reference		Reference		Reference		Reference	
Hearing aids (−)	0.355 (0.124, 1.015)	0.053	0.253 (0.081, 0.796)	0.019	0.277 (0.086, 0.889)	0.031	0.233 (0.066, 0.823)	0.024
Gender								
Female	Reference		Reference		Reference		Reference	
Male	1.339 (0.509, 3.518)	0.554	1.433 (0.513, 4.000)	0.492	1.146 (0.336, 3.915)	0.828	0.874 (0.240, 3.182)	0.838
Education								
Primary school	Reference		Reference		Reference		Reference	
Middle school	0.567 (0.129, 20,488)	0.452	0.469 (0.098, 2.257)	0.345	0.440 (0.090, 2.158)	0.312	0.332 (0.060, 1.856)	0.209
High school or GED	0.248 (0.058, 1.063)	0.060	0.230 (0.050, 1.051)	0.058	0.246 (0.053, 1.143)	0.074	0.208 (0.042, 1.023)	0.053
College or above	0.125 (0.026, 0.611)	0.010	0.126 (0.024, 0.657)	0.014	0.141 (0.026, 0.775)	0.024	0.101 (0.016, 0.619)	0.013
Hearing loss								
Mild + moderate	Reference		Reference		Reference		Reference	
Moderated Severe	0.823 (0.062, 10.841)	0.882	1.025 (0.078, 13.409)	0.985	1.217 (0.081, 18.237)	0.887	1.465 (0.092, 23.332)	0.787
Severe	0.459 (0.035, 6.015)	0.553	0.525 (0.040, 6.863)	0.623	0.616 (0.041, 9.175)	0.725	0.773 (0.049, 12.304)	0.855
Profound	0.213 (0.012, 3.922)	0.298	0.236 (0.012, 4.567)	0.340	0.253 (0.011, 5.654)	0.386	0.363 (0.015, 8.497)	0.529
Hypertension								
Hypertension (−)	Reference		Reference		Reference		Reference	
Hypertension (+)	2.004 (0.798, 5.033)	0.139	2.783 (1.017, 7.616)	0.046	2.905 (1.017, 8.299)	0.047	0.363 (0.015, 8.396)	2.914

OR, odds ratio; CI, confidence interval; GED, general equivalency diploma.

Model 1, unadjusted; Model 2, Model 1 + only adjusted age; Model 3, Model 2 + adjusted for living habits, including living alone, smoking and alcohol status; Model 4, Model 3 + adjusted for chronic disease, including diabetes and depressive symptoms.

While the educational levels were similar between HA+ and HA− groups in demographic and clinical characteristics, higher education was independently correlated with improved performance in delayed recall and spatial orientation in multivariate logistic models, even after adjusting for all covariates.

## 4 Discussion

This study assessed how HAs affect cognitive function in patients with ARHL, particularly delayed recall and spatial orientation. Adjusted logistic regression models revealed that HA use and higher educational levels were independently associated with better cognitive function, especially in these areas. Males were more prone to impaired delayed recall, while hypertension was associated with worse spatial orientation.

Our findings revealed that HA + had notably better cognitive function than HA-, especially in delayed recall, spatial orientation, language, attention, abstraction, and naming. This finding supports previous meta-analyses linking ARHL to cognitive decline [OR = 1.85, 95% CI = (1.59, 2.17)] and dementia [OR = 1.89, 95% CI = (1.50, 2.38)] (13). Additionally, HAs have been shown to lower the risk of cognitive decline, improve MoCA, enhance delayed verbal recall, and contribute to better postural balance, underscoring its protective cognitive benefits (14–16).

In this study, a discrepancy was observed between the orientation subdomains of the MoCA and MMSE tests. MoCA-orientation showed no significant group differences, while MMSE indicated better spatial orientation in the HA+ group but not temporal orientation. This difference may be due to the MoCA’s combined temporal and spatial orientation scale, leading to ceiling effects and reduced sensitivity (HA+: 5.532 vs. HA−: 5.316). In contrast, MMSE separated these orientations, allowing for more precise detection of changes. Our findings suggested that ARHL without HAs may specifically affect spatial orientation rather than temporal orientation, a distinction obscured by MoCA’s combined assessment.

This study found that the HA+ group had worse hearing but better cognitive outcomes than those not using them (HA–), suggesting a positive association between HA use and cognitive function in ARHL. These observed associations may be subject to reverse causality, wherein individuals with better cognition or higher education might be more likely to adopt or continue HA use. However, no significant educational differences were found between the groups in our study. Older adults over 80, those with more than 7 years of education, and those managing finances independently were more likely to use HAs (17). Negative attitudes toward hearing loss and social withdrawal were linked to discontinuing HA use (18). These findings highlighted the importance of psychosocial and behavioral factors in HA use and cognitive preservation, underscoring the need for interventions promoting positive attitudes and long-term use.

Our study found that the HAs and higher educational level were independently associated with better delayed recall, while male sex was independently associated with poorer delayed recall decline even after accounting for other variables. This aligns with previous research linking ARHL to declines in delayed recall and mental flexibility (19). Poor self-reported hearing was associated with a significant decline in delayed recall (*Δ* = −0.47, SE = 0.16, *p* < 0.01), and moderate-to-severe HL was negatively related to delayed verbal recall (*β* = −10.6, *p* = 0.019) (20, 21). Our findings indicated that delayed recall improved significantly with HAs. Previous studies supported this, showing that auditory rehabilitation through HAs or cochlear implants can enhance delayed recall performance (22).

Our study found that HA use and higher education were linked to better spatial orientation, while hypertension was associated with poorer abilities. This suggested that HAs not only enhanced hearing but also supported cognitive functions such as spatial orientation, improving dual-task gait performance in ARHL (23). Previous research supported that HAs improved gait step time, visuospatial working memory, and reduced fall risk, highlighting their clinical value in addressing disorientation in ARHL (24, 25).

Our study found no significant differences in educational levels between the two groups, suggesting comparability in demographic and clinical characteristics. Both HA use and higher education emerged as independent factors associated with better delayed recall and spatial orientation. Mohammad et al. (26) reported that a higher educational level correlated with greater cognitive reserve, which was associated with improved episodic memory in older adults. Similarly, Zarantonello et al. (27) found that a higher educational level (10 years) and increased cognitive reserve positively influence the accuracy of visuospatial working memory across the adult lifespan. This suggested that older adults with higher educational levels may possess greater cognitive reserve, enhancing their capacity to manage age-related cognitive decline and resist neuropathological changes before the onset of dementia symptoms (3). Notably, this protective effect may endure even when educational levels are evenly distributed across groups.

Our findings indicated cognitive decline in patients with ARHL without HAs. This could be related to reduced cortical thickness in the left para-hippocampal cortex (28). Our previous study showed weakened functional connectivity between the superior frontal gyrus (SFG) and cingulate gyrus (CG) in patients with ARHL, possibly as a compensatory response to impaired hearing. There was increased alpha activity in the left temporal lobe and improved SFG-CG connectivity after HAs, which may partly explain why HAs might restore cognitive reserve balance through neuroplasticity (29).

This study has some limitations, including a small sample size and a cross-sectional design, which were insufficient to clarify the relationship between cognitive improvement and HAs in ARHL. Even after accounting for multiple covariates, the possibility of residual confounding from unmeasured or inaccurately measured variables cannot be completely ruled out. To establish a causal relationship between HA use and cognitive outcomes, larger longitudinal RCTs are necessary. The wide 95% CI in the logistic regression indicated limited statistical power and potential parameter instability, likely due to the modest sample size and few outcome events. Multivariable logistic regression analyses targeted delayed recall and spatial orientation. Future research should involve larger, more balanced samples to explore additional cognitive domains and achieve more precise effect estimates.

## 5 Conclusion

Our study found that HA + participants had better cognitive function than those without (HA-) ARHL, particularly in delayed recall, spatial orientation, attention, naming, abstraction, and language and praxis, even among those with worse hearing and speech thresholds. After adjusting for various factors, logistic regression models showed that HA and a higher educational level decreased the risk of impaired delayed recall and spatial orientation. Males were more prone to reduced delayed recall, and hypertension was associated with poorer spatial orientation.

## Data Availability

The raw data supporting the conclusions of this article will be made available by the authors, without undue reservation.
