# Supplementary material for: The protective role of hearing aids on delayed recall and spatial orientation in age-related hearing loss
Source: Front Public Health. 2026 Mar 9;14:1753247. doi: 10.3389/fpubh.2026.1753247 (PMC13006218; doi:10.3389/fpubh.2026.1753247)
Supplement: Supplementary file 1 [file Table_1.docx]

**Supplementary Table 1 MoCA and MMSE sub-item between HA+/- groups. M(SD)**

|  | HA+ (n=47) | HA- (n=57) | Z | *p* |
| --- | --- | --- | --- | --- |
| MoCA |  |  |  |  |
| Delayed recall | 1.638 (0.062) | 0.754 (1.199) | -2.653 | 0.008** |
| Visuospatial and Executive Function | 3.979 (0.134) | 3.123 (0.167) | -3.566 | 0.000*** |
| Language | 2.532 (0.095) | 2.156 (0.102) | -2.673 | 0.008** |
| Attention | 5.702 (0.105) | 5.333 (0.123) | -2.478 | 0.013* |
| Naming | 2.723 (0.726) | 2.368 (0.924) | -2.776 | 0.005** |
| Abstraction | 1.638 (0.883) | 1.158 (0.109) | -3.138 | 0.002** |
| Orientation | 5.532 (0.904) | 5.316 (0.798) | -1.951 | 0.051 |
| MMSE |  |  |  |  |
| Spatial Orientation | 3.636 (0.141) | 3.175 (0.137) | -3.694 | 0.000*** |
| Temporal Orientation | 4.830 (0.555) | 4.790 (0.545) | -0.517 | 0.605 |
| Recall | 1.468 (0.149) | 0.930 (0.148) | -2.629 | 0.009** |
| Registration | 2.979 (0.213) | 2.790 (0.740) | -2.352 | 0.019* |
| Attention and Calculation | 4.745 (0.936) | 4.211 (0.166) | -2.417 | 0.016* |
| Language and Praxis | 7.936 (0.165) | 7.351 (0.152) | -2.795 | 0.005** |

HA+, age-related hearing loss with hearing aid; HA-, age-related hearing loss without hearing aid;

MoCA, Montreal Cognitive Assessment ; MMSE, Mini-Mental State Examination; **p*＜0.05; ***p*＜0.01; ****p*＜0.001.
